# The study for asthma in older adults: a combined analysis of the effects of occupational asthmagens, high body-mass index and smoking

**DOI:** 10.3389/fpubh.2025.1625623

**Published:** 2025-07-23

**Authors:** Jia-Qi Wang, Yuan-Yu Liang, Zhong-Xue Zhao, Wei Xie, Wan-Ning Sun, Ji-Yu Zou, Xiao-Dong Lv, Youfu He, Li-Jian Pang

**Affiliations:** ^1^The First Clinical College, Liaoning University of Traditional Chinese Medicine, Shenyang, Liaoning, China; ^2^Department of Pulmonology II, Affiliated Hospital of Liaoning University of Traditional Chinese Medicine, Shenyang, Liaoning, China; ^3^Tianjin Key Laboratory of Exercise Physiology and Sports Medicine, Institute of Sport, Exercise and Health, Tianjin University of Sport, Tianjin, China; ^4^Department of Cardiology, Guizhou Provincial People's Hospital, Guiyang, China

**Keywords:** asthma burden, high body-mass index, smoking, occupational asthmagens, older adults

## Abstract

**Background:**

Asthma remains a significant public health challenge globally, particularly among older adults who face diagnostic complexity, atypical symptom profiles, and frequent comorbidities. Despite global advances in asthma control, little attention has been paid to the evolving composition and geographic disparity of modifiable risk factors in this age group.

**Methods:**

We utilized data from the Global Burden of Disease Study 2021 to evaluate the long-term trends (1990–2021) in asthma burden attributable to high body-mass index (BMI), smoking, and occupational asthmagens among adults aged 60 years and older. Key metrics included age-standardized mortality rates (ASMR), age-standardized DALY rates (ASDR), and estimated annual percentage change (EAPC). Stratified analyses were conducted across Socio-demographic Index (SDI) quintiles, gender, and detailed 5-year age subgroups (60–64 to ≥95 years) to assess disparities across socioeconomic development, gender, and aging patterns, with additional stratification by geographic region.

**Results:**

From 1990 to 2021, asthma burden attributable to smoking and occupational asthmagens among older adults declined globally, while high BMI-related burden increased in several middle and low SDI regions. In terms of attribution proportion, the proportion of asthma deaths attributable to high BMI increased from 10.89 to 14.4%, with this upward trend being particularly pronounced in high SDI regions. Occupational asthmagens-related burden showed limited decline and remained elevated in low SDI regions. Gender-stratified analysis showed that the risk burden of smoking was higher in older men in Asia, whereas the high BMI-related burden was higher in women in more developed regions.

**Conclusion:**

This study demonstrates a structural shift in the composition of asthma risk factors among older adults, with high BMI emerging as a dominant contributor amid declining traditional behavioral risks such as smoking. There are significant differences among regions, age groups and genders. Targeted, region-specific strategies are essential to address these evolving risks and reduce inequities in asthma burden among aging populations.

## 1 Introduction

Asthma is a chronic inflammatory disease of the airways caused by the interaction of multiple genetic and environmental factors. It is characterized by a prolonged disease course, high heterogeneity, and complex management, substantially impairing patients’ quality of life and posing a significant socioeconomic burden (1). According to the Global Burden of Disease (GBD) estimates, asthma affected approximately 260 million people worldwide in 2021, resulting in 436,000 annual deaths, while the absolute number of asthma-related deaths continues to rise (2, 3). Although asthma is prevalent across all age groups, research and intervention efforts have disproportionately centered on pediatric and younger adult populations, leaving older adults understudied (4, 5).

Older adults (≥60 years) represent a critically understudied subgroup in asthma research. Atypical symptom presentation (e.g., overlapping manifestations with chronic obstructive pulmonary disease (COPD) and heart failure), high rates of misdiagnosis, and systematic exclusion from clinical trials have contributed to a paucity of evidence guiding geriatric asthma management (6, 7). Compounding these challenges, older patients frequently exhibit distinct risk profiles characterized by obesity-related metabolic dysregulation, prolonged exposure to environmental hazards (e.g., occupational asthmagens and tobacco smoke), and multimorbidity with hypertension, diabetes, and cardiovascular diseases (8, 9). These factors collectively drive poorer treatment responses and higher healthcare utilization compared to younger cohorts (10).

Mounting evidence suggests that modifiable risk factors play a pivotal role in determining asthma burden in older populations. High BMI, smoking, and occupational asthmagens (e.g., exposure to industrial dust and harmful gasses) have been identified as the three leading contributors to asthma-related mortality and disability. In 2019, high BMI alone accounted for 16.94% of global asthma-related disability-adjusted life years (DALYs), surpassing the shares attributable to smoking (9.87%) and occupational asthmagens (8.82%) (3, 11). These risk factors interact with biological aging processes and show distinct patterns by gender and region. For example, older males often have prolonged exposure histories to smoking and occupational pollutants, leading to elevated asthma mortality and reduced control, while older females may demonstrate greater vulnerability to smoke-related airway damage despite lower exposure levels (12–14).

Despite these insights, critical knowledge gaps persist. First, prior studies have predominantly examined individual risk factors in isolation, neglecting potential synergies between obesity, smoking, and occupational asthmagens in the context of aging-related physiological changes. Second, existing analyses lack granularity in dissecting how risk factor contributions evolve temporally and geographically, especially across gender-stratified subgroups and SDI tiers (15, 16). Third, limited data exist to inform targeted interventions for older adults, a population projected to represent 22% of the global asthma burden by 2040 (2).

To address these gaps, the present study utilizes data from the GBD 2021 study to analyze long-term trends in asthma burden among adults aged 60 years and older from 1990 to 2021. We systematically evaluate changes in ASMR and ASDR attributable to high BMI, smoking, and occupational asthmagens, and investigate their geographic distribution and associations with gender, age, and SDI. This study aims to inform more targeted and region-specific strategies for asthma prevention and control in the context of global population aging.

## 2 Methods

### 2.1 Data sources

The data for this study come from the latest GBD Study 2021 database, a large-scale international collaborative project led by the Institute for Health Metrics and Evaluation (IHME) at the University of Washington to systematically assess the global burden of disease and risk factors through a standardized methodology (17, 18). The study is to systematically assess the global health burden of disease and risk factors through the implementation of a standardized methodology. GBD 2021 covers the period 1990 to 2021, and covers 32 years of trends in the global burden of asthma disease. The data cover 21 GBD regions, such as Central Asia, North Africa and Middle East, Eastern Europe, and High-income North America, etc. The regional delineation is based on GBD’s standardized regional definitions and is globally comparable and representative. Meanwhile, all countries and regions of the world were categorized at five levels according to the SDI, including high, high-middle, middle, low-middle, and low SDI. Our study focused on the global burden of asthma disease in the older age group of 60 years and above. This age group was further subdivided into subgroups (e.g., 60–64, 65–69, 70–74, 75–79, 80–84, 85–89, 90–94, and 95 + years) to characterize the age heterogeneity of asthma burden within the older age group in more detail. The present study analyzed three main categories of external risk factors for asthma burden, High BMI, defined as BMI ≥ 25 kg/m^2^. Smoking, including active and passive smoking exposure, and Occupational Asthmagens, including industrial dust, fumes, and hazardous gas exposure.

To quantify asthma health loss in the older adult population, the following metrics were used in this study: the ASMR, ASDR, DALYs, Age-standardized rate (ASR), and EAPC. Through the elaboration of the above data sources, this study ensures the international comparability of the data and the reliability of the analysis results, and fully reflects the rigor and comprehensiveness of the analysis of the burden of disease of asthma from a global perspective.

### 2.2 Statistical analyses

Consistent definition and quantitative assessment of the burden of asthma and associated risk factors based on the standardized methodology of GBD 2021. ASMR represents the rate of asthma-related deaths per 100,000 population under a hypothetical standard population age structure to eliminate confounding by differences in population age structure across regions or time periods. ASDR represents the standardized rate of asthma-related DALYs per 100,000 population and is a core measure of the overall burden of chronic disease. DALYs is a composite indicator that is utilized for the assessment of the overall burden of non-fatal chronic diseases. It consists of two components, namely: Years of Life Lost denotes the number of years of life expectancy lost due to premature death from the disease, and Years Lived with Disability denotes the number of years of quality of life lost due to the disease state of asthma. Disability weights were assigned according to the severity of asthma. Taking into account the differences in the structure of the older adult population across regions, this study age standardized the ASMR and ASDR and used the standard population set by the GBD project as the weighting basis for calculations, in order to maintain comparability in cross-regional and cross-time analyses (19–21). The formula is $\mathrm{ASR}=\frac{\sum_{i=1}^{N}\;(\alpha_{i}\times W_{i})}{\sum_{i=1}^{N}\;W_{i}}$ , where $a_{i}$ indicates the age-specific rate in the ith age group, and $W_{i}$ denotes the weight of age group i in the GBD standardized population. The 95% uncertainty intervals (UIs) were determined as the 25th and 975th values among the ordered 1,000 draws (22). In order to quantify trends in the burden of asthma over time, the EAPC was utilized to assess the direction and magnitude of changes in ASMR and ASDR over time. The EAPC was calculated based on a log-linear regression model of the following form: ln(ASR)=α+β×year+ε , β is the slope of the regression line, year denotes the calendar year, ε is the residual error. The EAPC value is derived from the following formula: $\text{EAPC}=100\times (e^{\beta }-1)$ . An upward trend is considered significant when all 95% confidence intervals (CIs) for EAPCs are greater than 0. A decline in the observed values was evident when all CIs were less than 0, and a stable trend was identified when CIs crossed 0. The EAPC estimates facilitate the identification of the rate and direction of change in the burden of asthma over time, thereby enabling the development of intervention strategies that are responsive to these changes (23, 24). All statistical analyses were conducted using R software (version 4.3.2).

## 3 Results

### 3.1 Global burden and trends of asthma in the older attributable to high body-mass index, smoking, and occupational asthmagens

**Figure 1:**
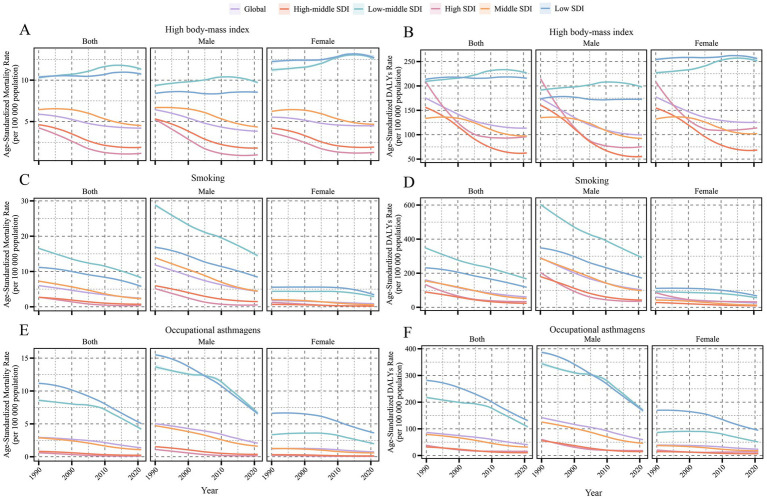
ASMR and DALY rate of asthma attributable to risk factors from 1990 to 2021, categorized by global and SDI regions. **(A)** ASMR due to high BMI. **(B)** ASDR due to high BMI. **(C)** ASMR due to smoking. **(D)** ASDR due to smoking. **(E)** ASMR due to occupational asthmagens. **(F)** ASDR due to occupational asthmagens.

From 1990 to 2021, the global asthma burden in the older adult population aged 60 years and older showed a risk-specific downward trend. The decline in smoking-related asthma burden was the most significant, with the smoking-related ASMR (per 100,000) of the global asthma burden in the older adult decreasing from 5.96 (95% UI: 0.69–12.39) to 2.41 (0.26–5.00), with an EAPC of −3.00 (95% CI: −3.07 to −2.93), The ASDR (per 100,000) also significantly dropped from 160.45 (95% UI: 18.50–312.91) to 60.89 (6.84–121.5), with an EAPC of −3.19 (95% CI: −3.26 to −3.13). Occupational asthma source control was the next most effective, with ASMR and ASDR decreasing to 1.42 (95% UI: 0.95–2.2) in 2021, with an EAPC of −2.44 (95% CI: −2.69–2.19), and 42.13 (95% UI: 29.2–61.72), with an EAPC of −2.41 (95% CI: −2.59–2.22). In contrast, the burden related to high BMI decreased at a slower pace, with the EAPC of ASMR (4.14 per 100,000) and ASDR (113 per 100,000) being only −1.26 (95% CI: −1.36 to 1.17) and −1.50 (95% CI: −1.63 to 1.36), respectively, reflecting the growing prominence of metabolic risk factors (Figures 1C–F).

When categorized by SDI, high and high-middle SDI regions showed systematic improvement, and the EAPC of the three risk factors ASDR was all less than −2.5 (Table 1). However, localized rebounds were observed; for example, in high SDI regions, ASDR attributable to high BMI exhibited a slight upward trend after 2015 (Figure 1). Meanwhile, in low and low-middle SDI regions, asthma burden associated with smoking and occupational asthmagens decreased notably. Smoking-related ASDR dropped from 230.93 per 100,000 (95% UI: 26.66–517.72) and 348.46 per 100,000 (95% UI: 40.15–780.93) in 1990 to 117.79 per 100,000 (95% UI: 12.37–259.37) and 167.78 per 100,000 (95% UI: 17.20–360.88) in 2021, respectively, corresponding to declines of 49.0 and 51.9%. Occupational asthmagens-related ASDR decreased by 51.8 and 48.3%, respectively, over the same period. Nevertheless, BMI-related ASDR in low and low-middle SDI regions showed an increasing trend, with EAPCs of +0.03 (95% CI: −0.02 to 0.07) and +0.38 (95% CI: 0.3 to 0.45), respectively (Figure 1; Table 1).

**Table 1:** ASMR, ASDR, and EAPC of asthma attributable to high BMI, smoking, and occupational asthmagens in older adults by SDI and region (1990–2021).

Location	High body-mass index				Occupational asthmagens				Smoking			
	ASMR	EAPC of ASMR	ASDR	EAPC of ASDR	ASMR	EAPC of ASMR	ASDR	EAPC of ASDR	ASMR	EAPC of ASMR	ASDR	EAPC of ASDR
Global	4.14 (1.74–6.74)	−1.26 (−1.36–1.17)	113 (48.63–179.65)	−1.5 (−1.63–1.36)	1.42 (0.95–2.2)	−2.44 (−2.69–2.19)	42.13 (29.2–61.72)	−2.41 (−2.59–2.22)	2.41 (0.26–5)	−3 (−3.07–2.93)	60.89 (6.84–121.5)	−3.19 (−3.26–3.13)
High SDI	1.1 (0.51–1.74)	−5.03 (−5.47–4.58)	95.04 (41.84–156.66)	−2.53 (−2.96–2.1)	0.1 (0.09–0.12)	−6.5 (−6.94–6.05)	15.56 (10.73–21.93)	−2.78 (−3.25–2.3)	0.35 (0.04–0.74)	−7.48 (−7.95–6.99)	32.66 (3.48–73.06)	−4.67 (−5.12–4.21)
High-middle SDI	1.83 (0.82–2.91)	−3.5 (−3.77–3.23)	62.39 (28.11–97.69)	−3.48 (−3.7–3.26)	0.23 (0.18–0.3)	−4.7 (−4.95–4.44)	10.54 (7.98–13.88)	−4.2 (−4.41–3.98)	0.7 (0.08–1.38)	−4.76 (−4.96–4.57)	23.43 (2.63–46.96)	−4.76 (−4.94–4.58)
Middle SDI	4.51 (1.98–7.21)	−1.42 (−1.58–1.26)	98.13 (42.18–154.16)	−1.34 (−1.49–1.19)	1.1 (0.86–1.41)	−3.45 (−3.67–3.23)	32.36 (25.19–41.15)	−3.27 (−3.47–3.08)	2.31 (0.27–4.53)	−3.9 (−4.07–3.73)	52.02 (5.97–101.15)	−3.79 (−3.93–3.66)
Low-middle SDI	11.17 (4.51–19.96)	0.46 (0.37–0.56)	224.46 (91.39–385.05)	0.38 (0.3–0.45)	4.46 (2.62–8.07)	−2.01 (−2.35–1.67)	113.23 (69.23–195.44)	−2.01 (−2.32–1.71)	8.29 (0.82–18.59)	−2.06 (−2.18–1.94)	167.78 (17.2–360.88)	−2.25 (−2.34–2.16)
Low SDI	10.53 (4.05–20.83)	0.18 (0.1–0.25)	213.36 (83.12–401.2)	0.03 (−0.02–0.07)	5.24 (2.86–9.18)	−2.52 (−2.75–2.28)	135.53 (77.33–226.45)	−2.48 (−2.69–2.27)	5.8 (0.59–13.41)	−1.96 (−2.13–1.79)	117.79 (12.37–259.37)	−2.17 (−2.31–2.03)
Western Europe	1.06 (0.46–1.66)	−4.98 (−5.54–4.43)	75.72 (32.91–124.95)	−3.68 (−3.96–3.39)	0.06 (0.05–0.07)	−5.38 (−6.07–4.7)	10.83 (7.48–15.26)	−2.47 (−2.96–1.97)	0.26 (0.03–0.57)	−7.51 (−8.11–6.9)	27.19 (2.8–60.33)	−5.24 (−5.6–4.87)
Central Europe	1.6 (0.73–2.51)	−5.72 (−6.25–5.19)	97.55 (42.87–159.13)	−4.04 (−4.41–3.66)	0.11 (0.09–0.13)	−8.23 (−8.96–7.49)	10.37 (7.12–15.09)	−5.88 (−6.55–5.21)	0.46 (0.05–0.95)	−7.29 (−7.79–6.8)	32.47 (3.51–69.12)	−5.27 (−5.69–4.84)
Eastern Europe	0.81 (0.37–1.24)	−7.49 (−7.98–6.99)	38.21 (17.07–60.25)	−6.32 (−6.68–5.95)	0.05 (0.04–0.07)	−8.27 (−8.8–7.73)	3.67 (2.63–5.18)	−6.42 (−6.78–6.06)	0.17 (0.02–0.34)	−9.18 (−9.85–8.5)	9.11 (1.09–18.36)	−7.81 (−8.32–7.3)
East Asia	1.72 (0.77–2.84)	−2.55 (−2.77–2.32)	39.77 (17.59–64.17)	−2.28 (−2.45–2.11)	0.34 (0.23–0.49)	−5.57 (−5.87–5.27)	12.4 (8.53–17.33)	−5.01 (−5.26–4.76)	1.18 (0.13–2.32)	−4.69 (−4.86–4.53)	28.6 (3.31–54.97)	−4.5 (−4.61–4.4)
Central Asia	7.8 (3.53–12.29)	−3.09 (−3.62–2.56)	193.24 (87.14–299.16)	−2.83 (−3.28–2.38)	0.91 (0.66–1.21)	−4.84 (−5.54–4.13)	31.42 (22.56–42.19)	−4.21 (−4.78–3.63)	1.81 (0.21–3.67)	−3.48 (−3.92–3.03)	46.58 (5.23–93.9)	−3.33 (−3.75–2.91)
South Asia	11.37 (4.49–21.75)	1.24 (1.07–1.41)	221.62 (88.13–405.15)	1.07 (0.93–1.21)	4.3 (2.36–8.73)	−2.51 (−2.9–2.12)	108.45 (61.87–210.04)	−2.52 (−2.87–2.16)	8.59 (0.82–19.97)	−2.26 (−2.42–2.1)	171.73 (16.88–384.74)	−2.49 (−2.6–2.37)
Southeast Asia	7.25 (3.19–11.91)	−0.42 (−0.57–0.27)	148.94 (63.38–242)	−0.42 (−0.56–0.29)	3.77 (2.8–5)	−1.8 (−1.96–1.65)	97.95 (73.57–129.07)	−1.71 (−1.86–1.57)	7.04 (0.81–13.62)	−3.11 (−3.28–2.94)	144.61 (17.03–278.41)	−3.05 (−3.19–2.91)
High-income North America	0.8 (0.37–1.23)	−3.5 (−3.83–3.17)	148.68 (65.19–250.45)	0.55 (−0.09–1.2)	0.09 (0.07–0.1)	−3.35 (−3.77–2.92)	23.17 (15.06–34.01)	1.01 (0.35–1.68)	0.22 (0.02–0.5)	−5.12 (−5.53–4.71)	45.68 (4.45–107.24)	−1.01 (−1.68–0.33)
Tropical Latin America	1.61 (0.7–2.54)	−2.5 (−2.82–2.18)	51.88 (22.92–82.4)	−2.49 (−2.8–2.19)	0.18 (0.14–0.23)	−4.9 (−5.55–4.24)	8.6 (6.2–11.85)	−4.34 (−4.91–3.78)	0.43 (0.04–1.01)	−5.39 (−5.69–5.08)	16.24 (1.6–36.36)	−5.1 (−5.41–4.79)
Andean Latin America	1.12 (0.46–2.02)	−3.22 (−3.31–3.12)	33.69 (15.09–56.45)	−2.53 (−2.61–2.44)	0.27 (0.17–0.39)	−3.36 (−3.53–3.18)	10.7 (7.68–14.63)	−2.4 (−2.56–2.24)	0.22 (0.02–0.57)	−4.9 (−5.1–4.69)	6.73 (0.66–15.84)	−4.15 (−4.33–3.96)
Central Latin America	1.72 (0.79–2.7)	−5.49 (−5.69–5.28)	47.62 (21.94–74.15)	−4.75 (−4.95–4.55)	0.24 (0.2–0.3)	−6.77 (−7.01–6.53)	9.73 (7.52–12.41)	−5.74 (−5.97–5.51)	0.28 (0.03–0.62)	−8.18 (−8.45–7.92)	8.44 (0.94–18.07)	−7.27 (−7.52–7.03)
Southern Latin America	1.56 (0.7–2.42)	−2.63 (−2.98–2.28)	149.81 (66.69–245.64)	−1.37 (−1.55–1.2)	0.15 (0.12–0.18)	−4.1 (−4.29–3.9)	24.41 (16.62–34.93)	−1.99 (−2.1–1.87)	0.3 (0.03–0.68)	−4.57 (−4.95–4.19)	38.77 (3.82–88.36)	−2.86 (−3.07–2.65)
Central Sub-Saharan Africa	15.54 (4.87–47.1)	0.84 (0.78–0.9)	306.26 (101.99–839.65)	0.7 (0.64–0.77)	6.18 (2.94–14.82)	−2.19 (−2.35–2.03)	152.18 (78.74–338.36)	−2.16 (−2.31–2.01)	2.04 (0.19–4.87)	−2.19 (−2.3–2.07)	47.39 (4.53–108.68)	−2.11 (−2.22–1.99)
Eastern Sub-Saharan Africa	6.52 (2.38–14.16)	−0.03 (−0.07–0.01)	142.82 (54.18–285.11)	0 (−0.05–0.04)	4.98 (2.71–8.99)	−2.48 (−2.58–2.38)	134.43 (76.71–226.62)	−2.33 (−2.43–2.24)	2.32 (0.24–5.06)	−2.88 (−2.97–2.79)	54.98 (5.92–115.13)	−2.69 (−2.77–2.6)
Western Sub-Saharan Africa	9.94 (4.31–16.79)	−0.27 (−0.34–0.2)	215.53 (91.48–352.32)	−0.26 (−0.34–0.18)	4.03 (2.6–5.93)	−2.37 (−2.46–2.27)	112.21 (74.19–160.41)	−2.24 (−2.3–2.17)	1.37 (0.13–3.06)	−2.37 (−2.43–2.3)	31.51 (3.04–68.58)	−2.34 (−2.4–2.28)
Southern Sub-Saharan Africa	21.87 (9.8–35.14)	0.17 (−0.4–0.74)	404.98 (182.8–644.58)	0.19 (−0.36–0.76)	3.4 (2. 26–4.85)	−0.9 (−1.46–0.34)	82.25 (56–115.3)	−0.87 (−1.42–0.32)	4.58 (0.5–9.63)	−2.98 (−3.42–2.55)	96.69 (10.58–199.24)	−2.65 (−3.08–2.23)
North Africa and Middle East	12.62 (5.59–20.12)	−1.78 (−1.89–1.66)	277.08 (126.06–424.34)	−1.67 (−1.77–1.57)	0.99 (0.72–1.31)	−4.1 (−4.33–3.86)	28.93 (21.66–37.26)	−3.85 (−4.07–3.62)	2.86 (0.3–5.97)	−3.57 (−3.67–3.47)	65.29 (6.82–131.93)	−3.46 (−3.56–3.36)
Australasia	1.67 (0.75–2.63)	−3.73 (−4.56–2.89)	100.08 (44.44–161.22)	−2.73 (−2.99–2.46)	0.12 (0.09–0.15)	−4.37 (−5.26–3.48)	14.31 (10.04–20.09)	−1.94 (−2.17–1.7)	0.31 (0.03–0.75)	−6.33 (−7.21–5.45)	22.26 (2.12–52.26)	−5.17 (−5.49–4.85)
High-income Asia Pacific	0.66 (0.28–1.13)	−7.84 (−8.34–7.34)	30.32 (12.95–50.53)	−6.5 (−6.9–6.11)	0.13 (0.09–0.17)	−9.55 (−10.04–9.06)	11.24 (7.82–15.79)	−7 (−7.33–6.67)	0.33 (0.04–0.74)	−10.48 (−11.03–9.94)	15.84 (1.65–34.41)	−8.94 (−9.35–8.52)
Caribbean	3.12 (1.36–5.3)	−1.26 (−1.52–1.01)	91.6 8 (40.39–147.16)	−1.03 (−1.21–0.85)	0.83 (0.5–1.51)	−0.65 (−0.75–0.55)	27.06 (18.02– 44.43)	−0.55 (−0.63–0.46)	1.01 (0.11–2.28)	−2.85 (−3.09–2.62)	29.95 (3.28–65.02)	−2.54 (−2.72–2.36)
Oceania	36.91 (15.11–69.74)	−0.88 (−0.97–0.79)	683.3 (282.78–1274.34)	−0.78 (−0.86–0.7)	6.3 2 (3.62–11.3)	0.08 (−0.02–0.18)	148.5 (87.01–262.52)	0.06 (−0.04–0.17)	11.74 (1.12–27)	−1.82 (−1.92–1.73)	249.3 (24.48–558.59)	−1.81 (−1.91–1.71)

Regionally, BMI-attributable asthma burden decreased in most regions; however, High-income North America and Southern Sub-Saharan Africa experienced rising ASDR trends in recent years. For example, the ASDR in Southern Sub-Saharan Africa reached 404.98 per 100,000 (95% UI: 182.8–644.58) in 2021, significantly above the global average. The EAPC for this region’s high BMI-related ASDR from 1990 onward was +0.19 (95% CI: −0.36 to 0.76) (Table 1).

Asia and Africa showed high smoking-related asthma burdens globally, yet varying trends emerged within Asia. High-income Asia Pacific experienced the fastest decline in smoking-related ASDR (EAPC: -8.94; 95% CI: −9.35 to −8.52). East Asia and Central Asia maintained relatively low smoking-related ASDR in 2021, at 28.6 per 100,000 (95% UI: 3.31–54.97) and 46.58 per 100,000 (95% UI: 5.23–93.9), respectively. However, Southeast Asia and South Asia remained among the highest globally, with ASDR at 144.61 per 100,000 (95% UI: 17.03–278.41) and 171.73 per 100,000 (EAPC: -2.49; 95% CI: −2.6 to −2.37), respectively.

**Figure 2:**
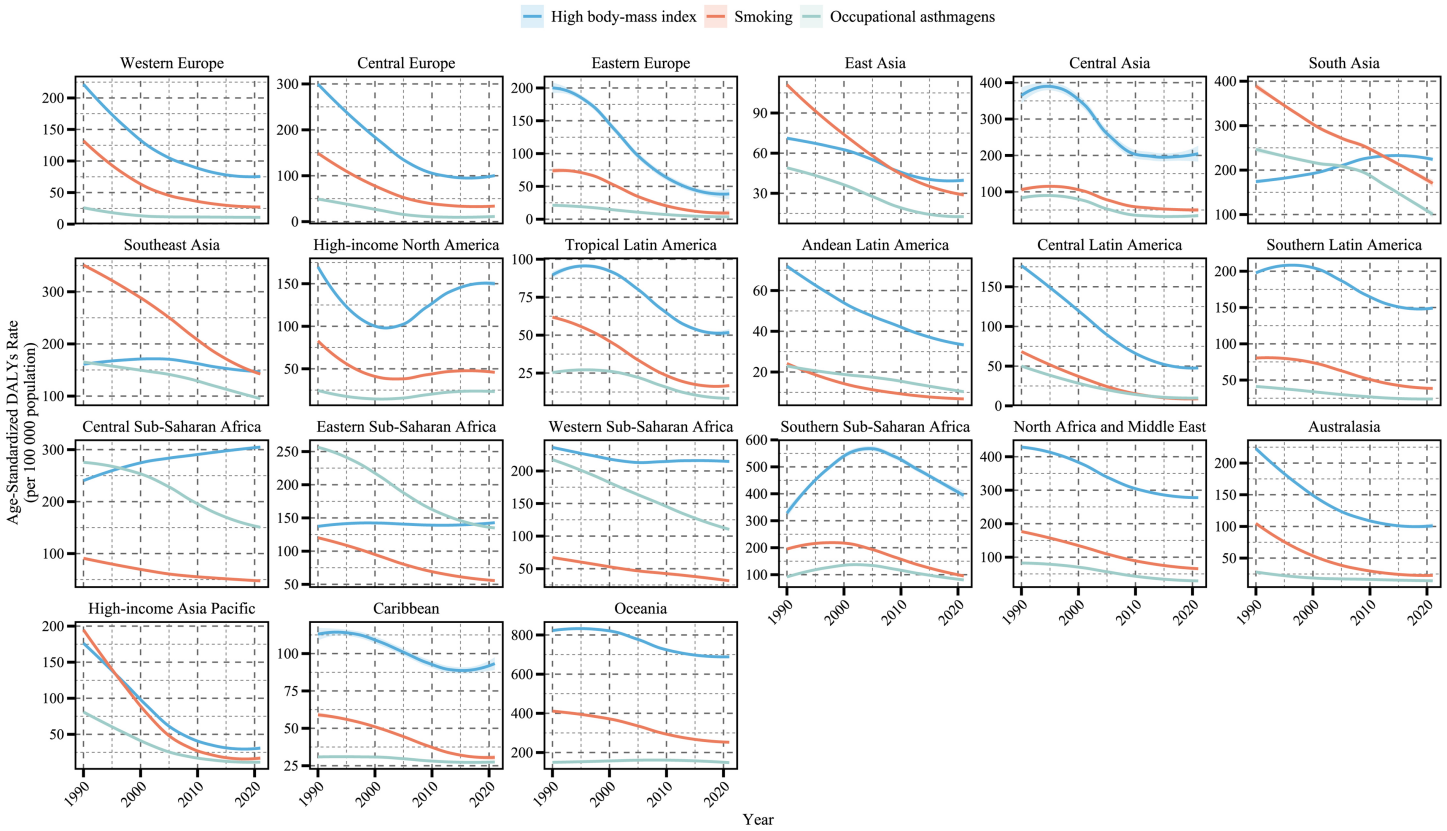
ASDR (per 100,000 population) of asthma attributable to risk factors from 1990 to 2021, across 21 GBD regions. The figure illustrates the contributions of high BMI (blue line), smoking (orange line), and occupational asthmagens (green line) to asthma ASDR.

The burden of occupational asthmagens-related asthma decreased significantly in Central Saharan Africa, Eastern Saharan Africa and South Asia, but there was no significant change in the burden of asthma in Oceania in recent years. However, it still maintained a high level, and its ASDR in 2021 was among the highest in the world (148.5 per 100,000; EAPC: +0.06, 95%CI:-0.04 to 0.17). It is noteworthy that although the occupational asthmage-related burden in High-income North America was among the lowest globally in 2021, with an ASDR of 23.17 per 100,000 (95%UI: 15.06–34.01), but the EAPC of ASDR was positive (1.01, 95% CI: 0.35–1.68) (Figure 2; Table 1), showing a slow upward trend. This anomaly may be related to the residual occupational asthmagens in some industries in the region, delayed onset of older adult workers and other factors (Figure 2; Table 1).

Therefore, the burden of asthma in the older adult population is characterized by an overall decline, structural change and regional disparities. The smoking- and occupational-related burdens exposure decreased significantly, while the high BMI-related burden continued to increase and gradually became the dominant risk in some low and medium SDI areas, which deserved close attention. Furthermore, occupational asthma prevention warrants continuous vigilance even in economically developed regions due to potential localized rebounds.

### 3.2 Spatial distribution of asthma-related mortality and disability burden in older adults

**Figure 3:**
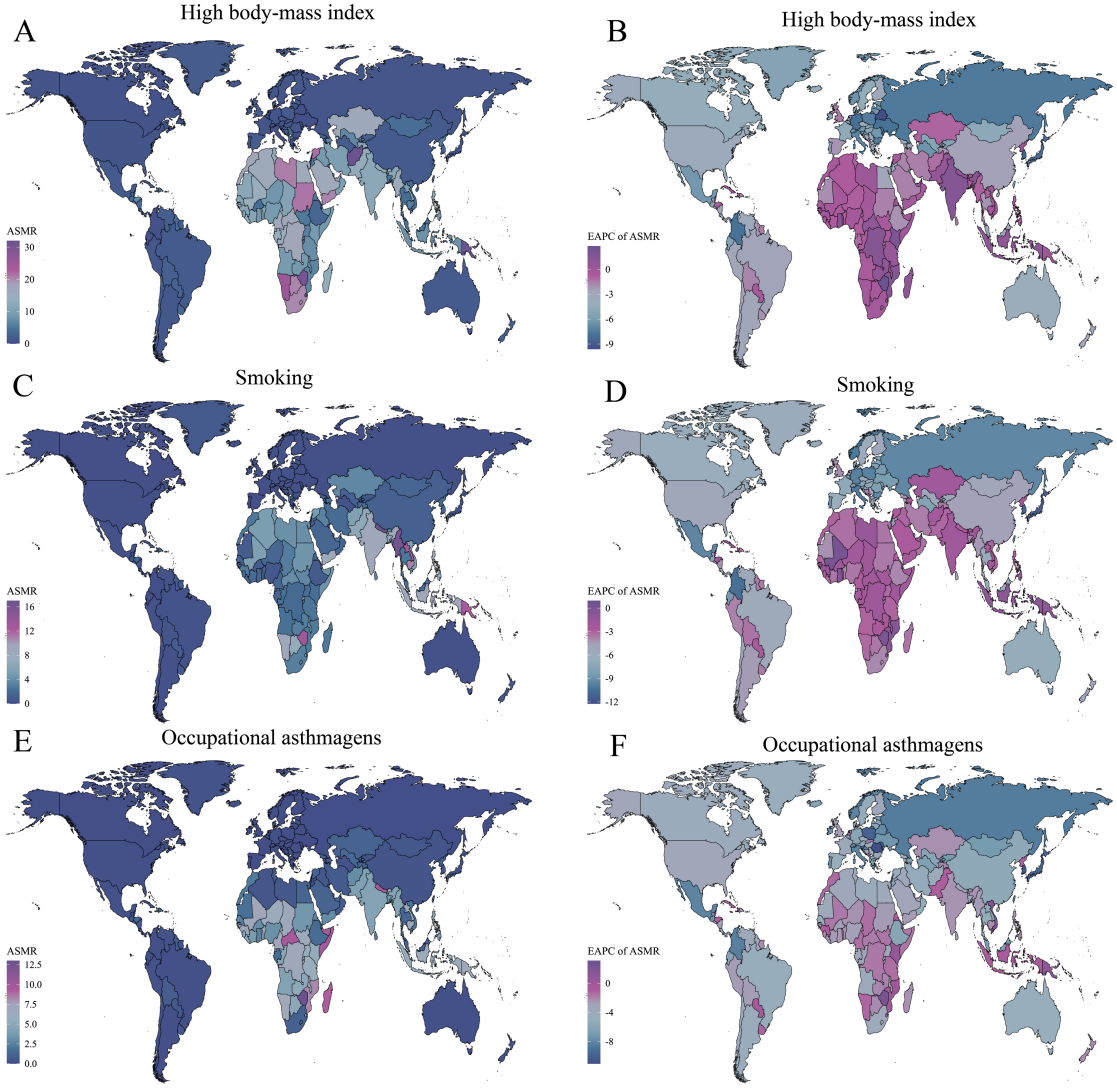
Global spatial distribution of ASMR and EAPC of asthma attributable to risk factors in 2021. **(A)** ASMR due to high BMI. **(B)** EAPC of ASMR due to high BMI. **(C)** ASMR due to smoking. **(D)** EAPC of ASMR due to smoking. **(E)** ASMR due to occupational asthmagens. **(F)** EAPC of ASMR due to occupational asthmagens.

**Figure 4:**
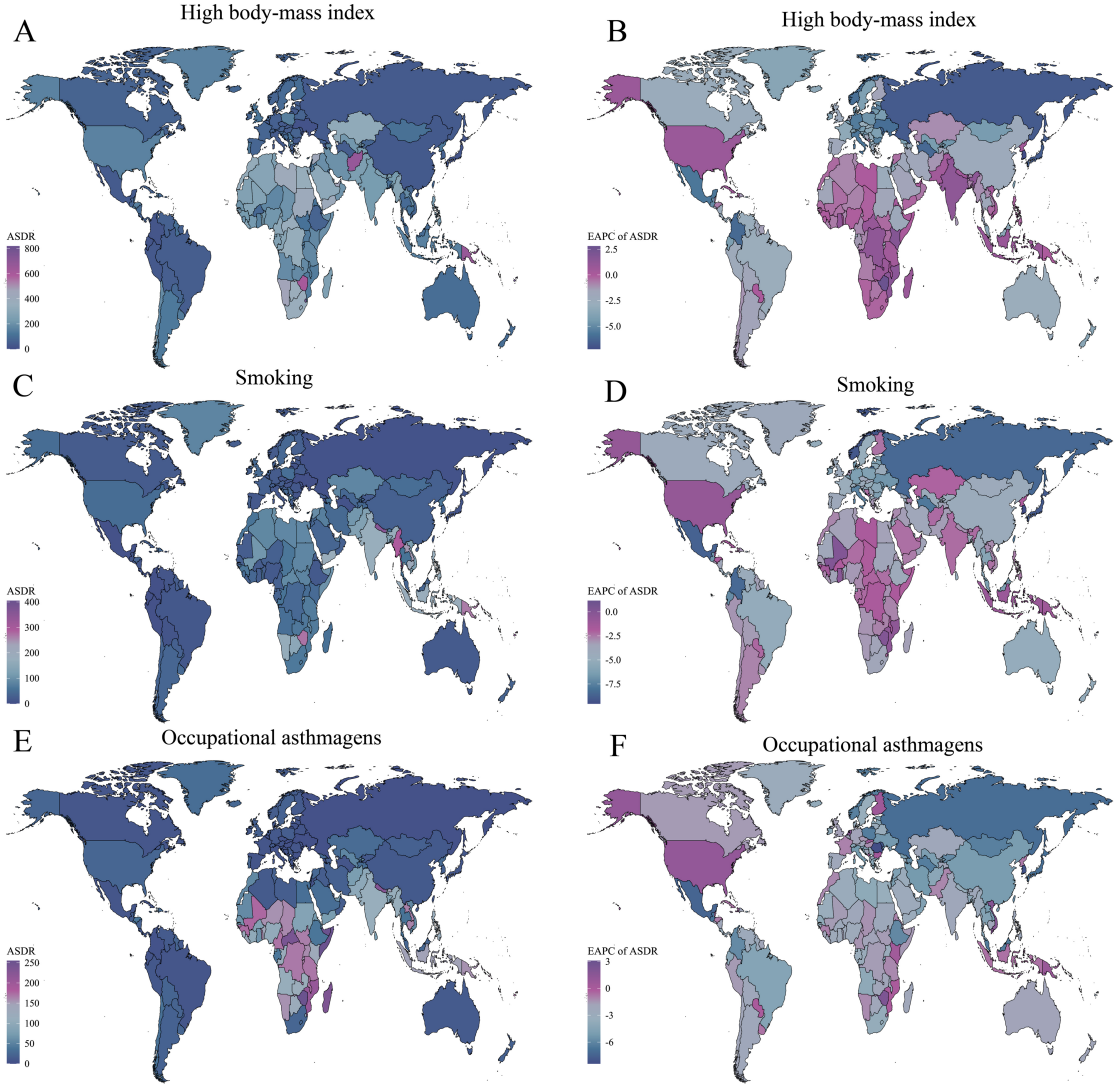
Global spatial distribution of ASDR and EAPC of asthma attributable to risk factors in 2021. **(A)** ASDR due to high BMI. **(B)** EAPC of ASDR due to high BMI. **(C)** ASDR due to smoking. **(D)** EAPC of ASDR due to smoking. **(E)** ASDR due to occupational asthmagens. **(F)** EAPC of ASDR due to occupational asthmagens.

In terms of spatial distribution, ASMR and ASDR attributable to high BMI were notably high among older adults in Central and Eastern Africa, parts of the Arabian Peninsula, and South Asia. Particularly in countries such as Yemen, Zimbabwe, Libya, and Afghanistan, ASMR exceeded 20 per 100,000, and ASDR surpassed 600 per 100,000 (Figures 3A, 4A). This highlights the significant threat of obesity-related asthma mortality and disability among older adult populations in socioeconomically disadvantaged regions. The EAPC trends indicated substantial declines in ASMR and ASDR in most high-income countries of Europe and North America (EAPC < −3), while positive or even increasing EAPC values were observed in underdeveloped regions, including Sub-Saharan Africa, South Asia, and Western Asia, reflecting a growing obesity burden and rising asthma risks among older adults in these areas (Figures 3B, 4B).

Regarding smoking-related burdens, regions with high ASMR and ASDR in 2021 were mainly concentrated in Southeast Asia (e.g., Laos and Myanmar) and parts of Africa. Smoking-attributable asthma ASMR exceeded 16 per 100,000 in some countries, and ASDR remained elevated in Southeast Asia and parts of South Asia, with notably high values observed in Laos (239.96 per 100,000, 95% UI: 25.77–522.61) and Myanmar (289.52 per 100,000, 95% UI: 29.54–637.07) (Figures 3C, 4C).

The spatial distribution of occupational asthmagens-related asthma burden among older adults also exhibited significant geographical disparities. ASMR and ASDR due to occupational asthmagens (e.g., industrial dust and harmful gasses) were notably high in economically underdeveloped regions, such as Africa, South Asia, and Southeast Asia. Remarkably, upward trends were observed in developed countries such as the Kingdom of the Netherlands and the United States of America, with ASDR-related EAPCs of +1.81 (95% CI: 1.35–2.28) and +1.18 (95% CI: 0.51–1.85), respectively (Figures 3D–F, 4D–F).

### 3.3 Age and gender disparities in asthma burden

**Figure 5:**
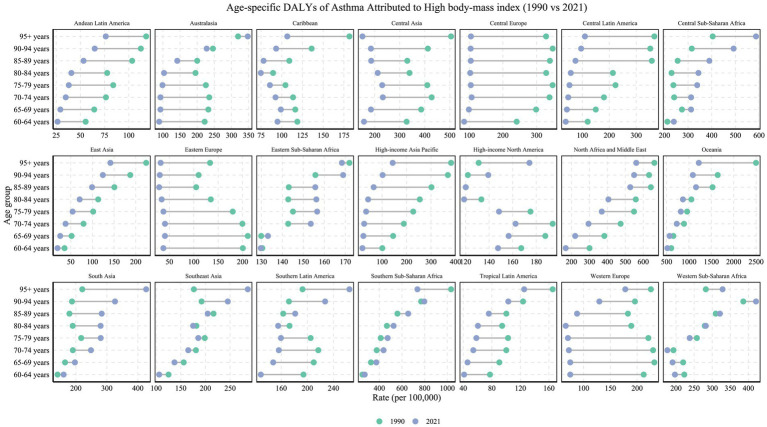
Age-specific DALYs rates (per 100,000 population) of asthma attributable to high BMI in 1990 versus 2021, across 21 GBD regions and various age groups. The figure illustrates the changes in DALY rates for each region across different age groups (1990 in green, 2021 in blue).

Globally, age-specific disparities in high BMI-related asthma DALYs among older adults were evident (Figure 5). In high-income regions such as Eastern Europe and High-income Asia Pacific, consistent declines were observed across all age groups from 60 to 84 years, with the 65–69 age group experiencing reductions of over 80%. In contrast, dramatic increases were seen among the oldest-old (≥90 years) in middle- and low-income regions such as South Asia and Southeast Asia. In South Asia, DALYs for individuals aged 90–94 years rose from 187.62 per 100,000 (95% UI: 68.47–394.34) in 1990 to 326.29 per 100,000 (95% UI: 121.04–678.27) in 2021. For those aged ≥95 years, DALYs surged from 220.77 per 100,000 (95% UI: 76.85–477.29) to 426.84 per 100,000 (95% UI: 148.55–944.32), marking a substantial 93.2% increase. Southeast Asia exhibited a similar upward pattern, with DALYs in the ≥95 age group increasing by 61.9% over the same period (Figure 5). Notably, in more developed regions such as High-income North America and Western Europe, older females recorded slightly higher ASMR and DALYs than males (sex ratio <1), which may reflect gender differences in obesity prevalence, fat distribution, and metabolic adaptation in later life (25–27).

**Figure 6:**
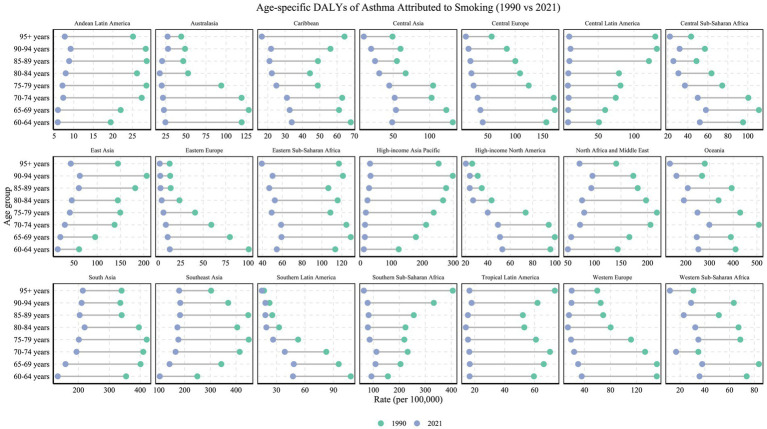
Age-specific DALYs rates (per 100,000 population) of asthma attributable to smoking in 1990 versus 2021, across 21 GBD regions and various age groups. The figure illustrates the changes in DALY rates for each region across different age groups (1990 in green, 2021 in blue).

In contrast to the clear age-related trends seen for high BMI, smoking-related DALYs among older adults did not exhibit significant age-specific variation but rather demonstrated strong regional disparities (Figure 6). In particular, regions such as South Asia (2021 ASDR: 171.73 per 100,000) and Southeast Asia (2021 ASDR: 144.61 per 100,000) consistently reported some of the highest burdens worldwide across all age groups (Table 1). Pronounced gender disparities were also observed, especially in Asia, where male-to-female mortality ratios due to smoking-related asthma reached high levels. For instance, in Central Asia, the mortality sex ratio for the 65–69 age group reached 26:1, while in East Asia, the same age group exhibited a ratio of 13:1. These figures underscore the longstanding gender gap in smoking behaviors, particularly in traditional societies of South and East Asia, where female smoking rates remain low. Over time, this disparity has led to significantly higher cumulative respiratory health risks for older males in these regions (28, 29).

**Figure 7:**
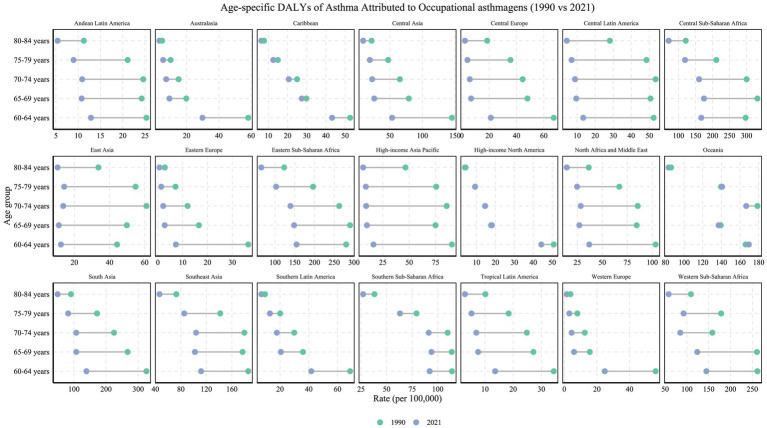
Age-specific DALYs rates (per 100,000 population) of asthma attributable to occupational asthmagens in 1990 versus 2021, across 21 GBD regions and various age groups. The figure illustrates the changes in DALY rates for each region across different age groups (1990 in green, 2021 in blue).

**Figure 8:**
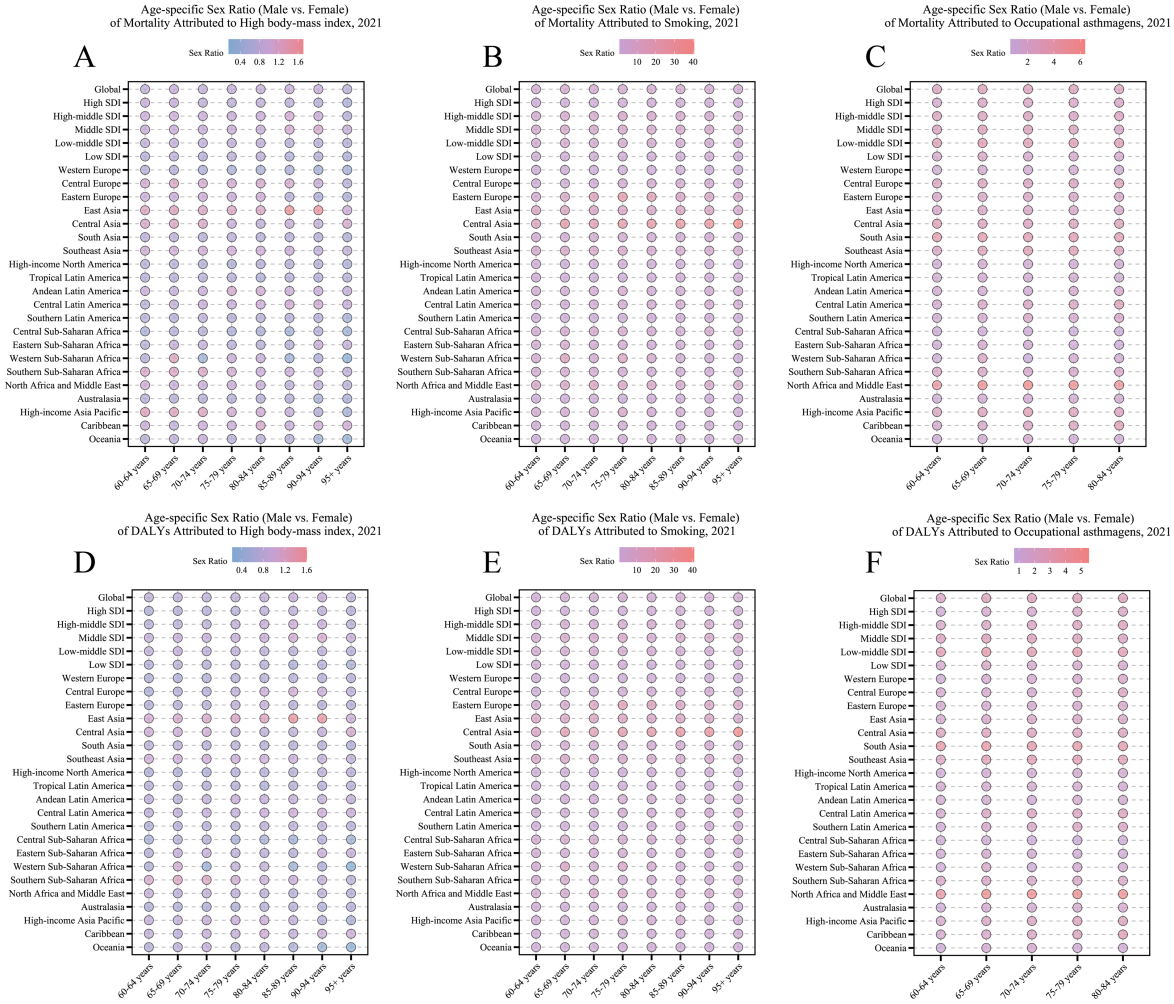
Age-specific sex ratio (male vs. female) of mortality and DALYs rates for asthma attributable to risk factors in 2021, across global, five SDI regions, and 21 GBD regions. **(A)** Age-specific sex ratio of mortality attributable to high BMI. **(B)** Age-specific sex ratio of mortality attributable to smoking. **(C)** Age-specific sex ratio of mortality attributable to occupational asthmagens. **(D)** Age-specific sex ratio of DALYs attributable to high BMI. **(E)** Age-specific sex ratio of DALYs attributable to smoking. **(F)** Age-specific sex ratio of DALYs attributable to occupational asthmagens.

Occupational asthmagens-related DALYs demonstrated a clear pattern of age clustering, with the heaviest burden concentrated in the 60–74 age group across most regions, including South Asia, Eastern Sub-Saharan Africa, and Central Sub-Saharan Africa. The highest values were typically found among individuals aged 60–64. Strikingly, in Oceania, the burden of occupational asthma showed virtually no decline across age groups since 1990, with persistently elevated ASDRs in the 60–64 (169.42 per 100,000 in 2021) and 70–74 age groups (166.63 per 100,000 in 2021) (Figure 7). In terms of gender differences, older males globally exhibited occupational asthmagens-related DALYs two to four times higher than females (Figure 8C). This disparity was especially pronounced in low-middle SDI regions; for example, in North Africa and the Middle East, the sex ratio reached 6:1 in the 65–69 age group. The overrepresentation of males in high-risk occupations such as mining and construction results in a disparately high burden of occupational asthmagens-related asthma. This pattern reflects significant regional variations in the gender composition of hazardous workforces and underscores the urgent need for enhanced labor protection policies in these settings. Further analysis of sex ratios across different risk factors revealed distinct regional and age-specific patterns. As shown in Figure 8A, the age-specific sex ratio of mortality attributable to high BMI varies considerably, with older females in some developed regions having a slightly higher risk than males. In contrast, Figure 8B demonstrates that mortality attributable to smoking is overwhelmingly higher among older males, particularly in Asia and Central Asia. Regarding DALYs, Figure 8D and Figure 8E show similar trends for high BMI and smoking, respectively, with gender differences persisting across age groups and regions. Notably, for occupational asthmagens, Figure 8F illustrates that the DALY burden is consistently higher in males, especially in regions with a predominance of male workers in high-risk occupations. These findings underscore the need for gender-specific prevention and intervention strategies targeting major modifiable risk factors for asthma in older adults.

### 3.4 Association between socio-demographic index and asthma burden

**Figure 9:**
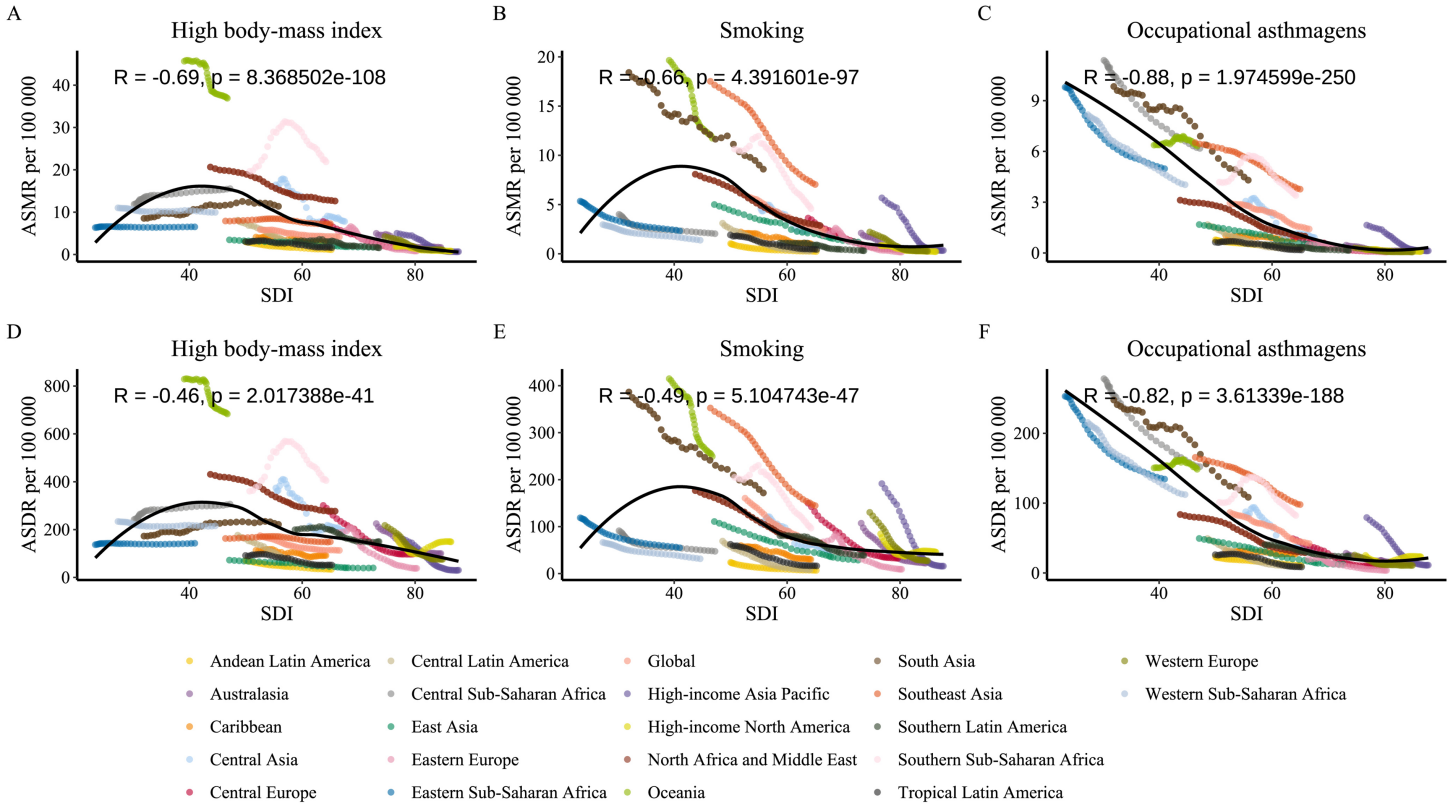
Correlation analysis between ASMR and ASDR of asthma attributable to risk factors and SDI from 1990 to 2021, across 21 GBD regions. The figure displays the correlation coefficient (R) and *p*-value. **(A)** Correlation between ASMR due to high BMI and SDI. **(B)** Correlation between ASMR due to smoking and SDI. **(C)** Correlation between ASMR due to occupational asthmagens and SDI. **(D)** Correlation between ASDR due to high BMI and SDI. **(E)** Correlation between ASDR due to smoking and SDI. **(F)** Correlation between ASDR due to occupational asthmagens and SDI.

Asthma-related ASMR and ASDR among older adults showed a generally inverse relationship with SDI across all three major risk factors. Regions with lower SDI were consistently associated with higher asthma burden. Among these factors, the correlation was strongest for occupational asthmagens (ASMR: *R* = −0.88; ASDR: *R* = −0.82; both *p* < 0.001), underscoring the continued challenges of occupational asthma prevention in lower-SDI settings (Figures 9C,F). In areas such as Central Sub-Saharan Africa, South Asia, and Oceania, occupational asthmagens remain key contributors to mortality and disability in older populations—likely due to limited labor protections and the economic reliance on resource-heavy sectors like mining, textiles, agriculture, and construction (30).

Smoking-related asthma burden also displayed significant negative correlations with SDI (ASMR: *R* = −0.66; ASDR: *R* = −0.49; both *p* < 0.001) (Figures 9B,E). However, several middle SDI regions, notably South Asia and parts of Oceania, deviated from the overall trend. These deviations may reflect persistent tobacco use among older males and gaps in tobacco control implementation.

In contrast, the association between BMI-related asthma burden and SDI was relatively weaker, exhibiting a nonlinear trend in certain regions. Although the correlation between BMI-related ASMR and SDI remained moderately negative (*R* = −0.69), the association with ASDR was somewhat less correlated (*R* = −0.46), with positive trends observed in countries with SDI between 50 and 60, such as Southern Sub-Saharan Africa and Central Asia (Figures 9A,D). This indicates that rapid urbanization and lifestyle transitions in certain middle-income countries are contributing to increasing obesity burdens among older adults, emerging as a dominant asthma risk factor. Conversely, developed countries, benefiting from improved obesity management and healthcare interventions, have seen reductions in BMI-related asthma burdens.

Our findings highlight the heterogeneous and partially nonlinear relationship between SDI and asthma burden among older adults, emphasizing the need to develop targeted strategies tailored to regional risk characteristics.

### 3.5 Shifting composition of risk factors for asthma burden among older adults (1990–2021)

Building on the SDI-linked risk divergences, significant changes occurred in the composition of major risk factors contributing to asthma mortality and disability among older adults globally and regionally from 1990 to 2021. The proportion attributable to high BMI generally increased; globally, the high BMI-related asthma burden in older adults rose from 13.15 to 16.69%, and the proportion of asthma deaths attributed to BMI increased from 10.89 to 14.4%. This trend was particularly notable in high SDI regions, highlighting obesity as an increasingly critical risk factor for asthma burden.

**Figure 10:**
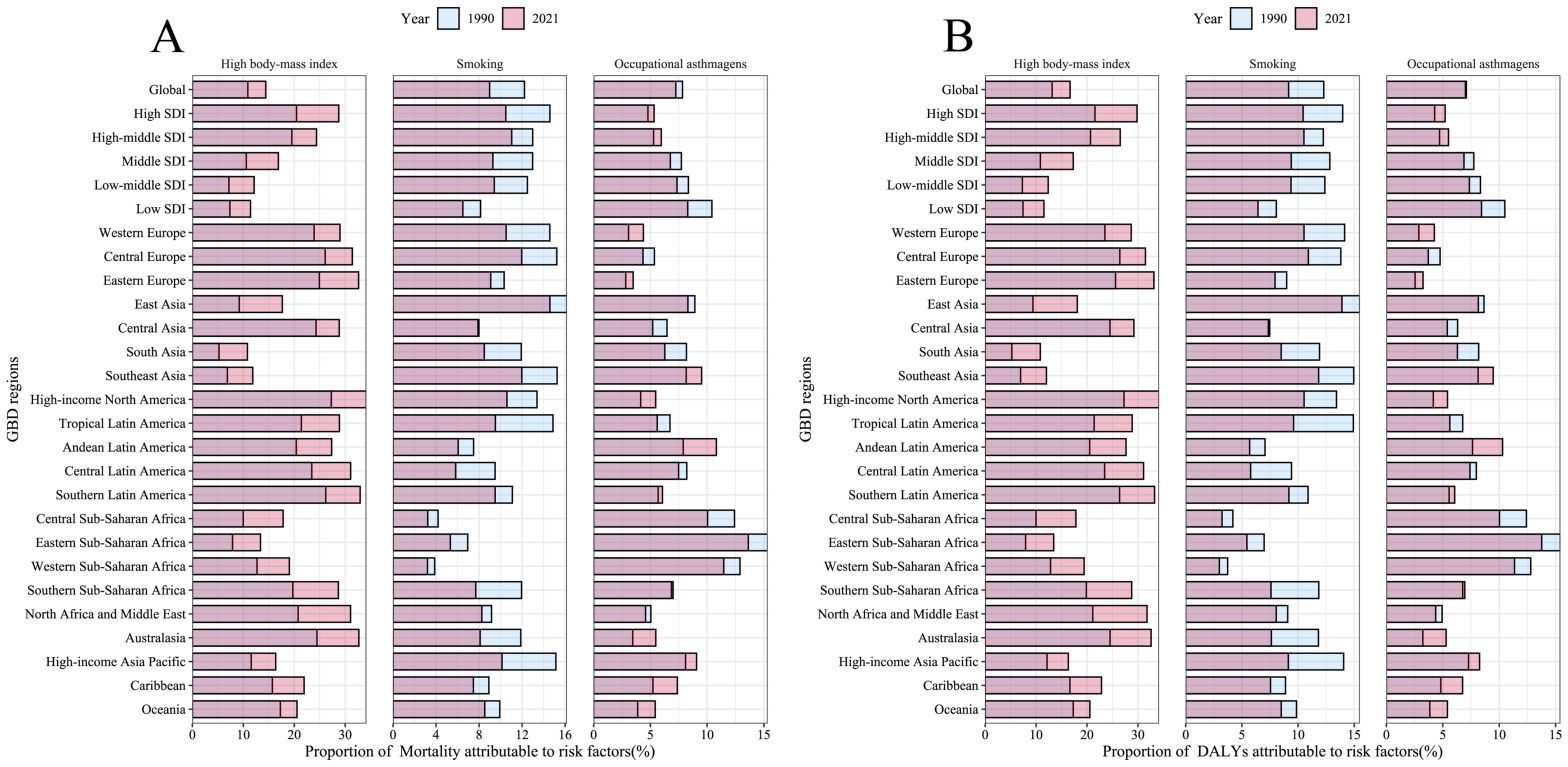
Proportion of asthma mortality and DALYs rates attributable to risk factors in 1990 and 2021, across global, five SDI regions, and 21 GBD regions. **(A)** Proportion of asthma mortality attributable to high BMI, smoking, and occupational asthmagens in 1990 and 2021. **(B)** Proportion of asthma DALYs rates attributable to high BMI, smoking, and occupational asthmagens in 1990 and 2021.

Conversely, the smoking-attributable proportion of asthma mortality declined globally, especially in economically developed regions. For example, in Western Europe, the proportion of asthma deaths attributed to smoking among older adults decreased from 14.58 to 10.51%, and in High-income Asia Pacific from 15.14 to 10.14%, representing declines exceeding 5 percentage points. However, in regions like East Asia, smoking remained one of the primary contributors to asthma-related mortality and disability in older adults (Figure 10).

The proportion attributed to occupational asthmagens showed relatively modest declines globally. In regions such as Africa and Asia, occupational asthmagens still accounted for a significant proportion of asthma-related deaths among older adults (e.g., Eastern Sub-Saharan Africa: 13.62%, Western Sub-Saharan Africa: 11.46%, Southeast Asia: 9.52%), underscoring the urgent need to strengthen workplace exposure prevention and occupational disease management. Overall, the risk factor composition for asthma is shifting from being tobacco-dominated toward obesity-related, yet occupational asthmagens remains an important burden source in developing countries.

## 4 Discussion

Based on the GBD 2021 database, this study systematically analyzed long-term trends in asthma burden among older adults globally from 1990 to 2021, specifically focusing on three modifiable risk factors: high BMI, smoking, and occupational asthmagens. Over the past three decades, the overall asthma burden among older adults showed risk-specific declining trends globally. While smoking and occupational asthmagens-related burdens significantly decreased, high BMI-attributable burdens demonstrated notable regional and structural increases. These changes illustrate the complex evolution of asthma burden in the context of global aging.

Our findings indicate a significant global decline in smoking-related asthma burden, with particularly notable reductions in high and high-middle SDI regions. This decline aligns closely with worldwide tobacco control policies, enhanced public awareness regarding smoking risks, and changing societal attitudes toward tobacco use. However, tobacco control progress has been uneven, especially in Asian regions. Persistent deficiencies in tobacco control implementation across South and Southeast Asia impede progress toward reducing the region’s disproportionately high burden of smoking-attributable asthma, as starkly evidenced in Laos where 99.2% of the population reports secondhand smoke exposure in public places (31). Concurrently, culturally embedded practices sustain tobacco use among adult males in Khmer, Lao, and Vietnamese communities through its symbolic role in status conveyance. This pattern aligns conclusively with our findings on gender-specific asthma burden attributable to smoking (32, 33). This necessitates urgent enforcement of targeted policies, primarily substantial tobacco tax reforms to deter youth initiation and comprehensive bans on all forms of tobacco advertising, promotion, and sponsorship.

Occupational asthmagens-related burden exhibited a similar overall decline but remained high in low SDI countries. Regions such as Central Sub-Saharan Africa, South Asia, and Oceania persistently ranked among the highest globally, largely due to these regions’ economic reliance on resource-intensive industries such as mining, construction, and textile manufacturing. The combination of labor-intensive, high-dust, and hazardous-gas exposure industries, coupled with inadequate occupational safety regulations, contributes to the persistent occupational asthmagens problem in these areas (30, 34). Additionally, the slight rebound observed in occupational asthma burden in some high-income countries, such as North America, deserves attention. This rebound may relate to delayed health impacts of cumulative occupational asthmagens among older workers and inadequate management of emerging occupational risks (35). Consequently, even in economically advanced regions, continued efforts to enhance occupational asthma prevention strategies are essential to address novel asthmagen risks emerging from rapidly evolving sectors such as 3D printing additive manufacturing and lithium-ion battery recycling.

Unlike smoking and occupational risks, BMI-related asthma burden demonstrated significant increases in some regions, progressively becoming a dominant health risk factor for older adults globally. This upward trend was particularly pronounced in middle and low SDI regions, notably South Asia and Sub-Saharan Africa. In Sub-Saharan Africa, rapid urbanization has created an obesogenic food environment dominated by energy-dense processed foods (36), while traditional dietary patterns are being eroded (37), and relatively inadequate local health systems have increased the burden of asthma (38). Interestingly, despite an overall decline in high-income countries, BMI-related asthma burdens were slightly higher among older females compared to older males in certain developed regions, such as High-income North America and Western Europe. In moderate-to-severe asthma populations, the convergence of advanced age, female gender, and obesity significantly intensifies asthma pathogenesis, particularly through aggravated peripheral airway dysfunction and elevated exacerbation rates (39). This epidemiologic pattern highlights the critical need for gender-specific obesity interventions and precision prevention protocols tailored to older women.

Spatial analyses revealed substantial regional disparities in asthma burden, with higher burdens primarily concentrated in socioeconomically disadvantaged regions, including Central and Eastern Africa, South Asia, and Southeast Asia. Such disparities are closely linked to inadequate healthcare resources, limited disease screening and management capacities, and underdeveloped chronic disease prevention infrastructures. Additionally, our study observed a slight upward trend in occupational asthma burden in developed countries, including the Kingdom of the Netherlands and the United States, potentially reflecting delayed effects of cumulative exposure among older workers in specific industries. Future health policies and labor protection measures should closely monitor these delayed effects and enhance occupational health surveillance to mitigate long-term impacts on the older adult.

Further analysis of the relationship between asthma burden and the SDI revealed significant inverse correlations, particularly prominent for occupational asthmagens and smoking. In contrast, the association between high BMI-related asthma burden and SDI was weaker and nonlinear in certain regions. This suggests that middle SDI countries face rapidly increasing obesity-driven asthma burdens due to socioeconomic development and nutritional transitions, representing a new challenge for global asthma prevention efforts. Targeted health education, dietary interventions, and physical activity programs should therefore be initiated early in these regions to prevent obesity-related risks from following patterns observed previously in developed nations.

Finally, this study underscores a shift in asthma burden risk composition among older adults globally, moving away from traditional behavioral risks such as smoking and occupational asthmagens toward metabolic risks such as obesity. Moreover, metabolic risk factors have been consistently associated with systemic diseases such as cardiovascular disorders (40), malignancies (41), and immune-related conditions (42) in established research, further enhancing the generalisability of this study’s findings. This shift reflects broader transformations in global health risk structures, characterized by declining traditional risks and rising emerging metabolic risks, especially pertinent in the context of global aging. Therefore, future global asthma prevention strategies must prioritize metabolic risk management and prevention, incorporating multidimensional approaches that consider regional disparities, gender sensitivities, and continued vigilance for occupational asthmagens to effectively protect respiratory health among older adults.

This study has limitations, including inherent uncertainties in the model-based GBD estimates, lack of individual-level data, and absence of quantitative analysis of interactions among risk factors. Future research should integrate individual-level data and focus on risk-factor interactions to further enhance the precision and effectiveness of intervention strategies.

## 5 Conclusion

This study constitutes the first systematic analysis elucidating the long-term independent and joint trajectories of three predominant modifiable risk factors—smoking, high BMI, and occupational asthmagens—on asthma burden among older adults (aged ≥60 years) globally from 1990 to 2021. Our findings reveal significant global declines in mortality and disability attributable to smoking and occupational asthmagens, contrasting with rising BMI-driven burdens in middle and high-SDI regions—signifying a pivotal transition from tobacco-dominated to obesity-centric risk paradigms.

Persistent health inequities across geographic regions, gender strata, and age cohorts necessitate tailored preventive strategies calibrated to local epidemiological contexts. Future public health initiatives must prioritize integrated interventions directed at high-risk populations, with particular emphasis on older adult cohorts in resource-constrained settings, to effectively mitigate the escalating burden of modifiable respiratory risk factors.

## Data Availability

The original contributions presented in the study are included in the article/supplementary material, further inquiries can be directed to the corresponding author.
